# The Evolution of Norovirus, the “Gastric Flu”

**DOI:** 10.1371/journal.pmed.0050042

**Published:** 2008-02-12

**Authors:** Ben Lopman, Maria Zambon, David W Brown

## Abstract

The authors discuss the implications of a new study that presents compelling data to show that norovirus evolution is driven by immune selection pressure.

Linguistically speaking, the predominant viral cause of gastroenteritis has been evolving. Once evocatively called *winter vomiting disease*, the pathogen's name has changed alongside improved scientific understanding. First called Norwalk virus (or Norwalk-like virus) in reference to the Ohio town where specimens from a school outbreak enabled the seminal work that first characterised the virus, it was later dubbed the Small Round Structured Virus following visualisation by electron microscopy. The International Committee on Taxonomy of Viruses later settled on the present name of “norovirus”, classifying it as a member of the *Caliciviridae* family based on both morphology and phylogeny. A new study published in this issue of *PLoS Medicine* suggests that the colloquial “gastric flu” may have been the most apt term of all [1]. When media reports call “norovirus” just “a fancy word for gastric flu”, they allude to similar seasonality and the lack of effective therapeutics for influenza and norovirus, but the analogy may run deeper [2]. There are parallels with respect to influenza and norovirus evolution and human immunity.

In the new study, Ralph Baric and colleagues present compelling data to demonstrate that norovirus evolution is driven by immune selection pressure. The domain of the exposed viral capsid protein that binds carbohydrates in the human gut evolves in the face of herd immunity. Histoblood group antigens (HBGAs), a heterogeneous group of related carbohydrates on mucosal surfaces, provide a “docking station” for noroviruses, and there is a large variety of such HBGAs. These sites are similar, but distinct, so as the capsid mutates and subtly changes its shape, it can still find new binding sites on the mucosal surface of the gut. The virus survives despite the build-up of immunity in the population because there is room in antigenic space for the virus to evolve and remain infectious. As with influenza, the predominant circulating norovirus may also change its antigenicity frequently, giving it the potential to cause pandemics and necessitate regular reformulation of vaccine.

**Linked Research Article**

This Perspective discusses the following new study published in *PLoS Medicine*:

Lindesmith LC, Donaldson EF, LoBue AD, Cannon JL, Zheng DP, et al. (2008) Mechanisms of GII.4 norovirus persistence in human populations. PLoS Med 5(2): e31. doi:10.1371/journal.pmed.0050031

Through phylogenetic analysis of norovirus isolates, Ralph Baric and colleagues show that new epidemic strains arise as the variety of available cellular receptors permits antigenic drift in the viral capsid.

## Norovirus Epidemiology

Noroviruses are the most commonly detected pathogen both in sporadic cases and outbreaks of gastroenteritis. They are particularly problematic in environments where groups congregate and infection can be rapidly transmitted through both faecal and vomitus routes. Outbreaks affect health care facilities worldwide, and may cause massive disruption to providing care, substantial economic loss, and, according to some reports, mortality in vulnerable patient populations [3–5]. As is typical of positive-sense single-stranded RNA viruses, noroviruses are diverse. There are two main genogroups affecting humans and approximately 15 genotypes within these groups, with substantial genetic heterogeneity between genogroups (60% divergence in the ORF2 major capsid protein) and genotypes within a genogroup (approximately 20%–30% divergence). At least since 1995 a single type—genotype II.4—has been the predominant circulating virus.

## Noroviruses' Escape from Population Immunity?

Some individuals, upon being challenged with norovirus infection, develop gastroenteritis, while others develop asymptomatic infection and some show no signs of infection at all. This points to a role of acquired immunity as well as innate resistance to infection. Indeed, individuals who genetically encode the enzyme FUT2 a-fucosyltransferase and are secretor-positive (i.e., they express HBGAs) are susceptible to Norwalk virus (a GI.1 virus) infection. Distinct binding patterns have been described for a range of other GI and GII strains, including GII.4. Acquired immunity is not thought to last until a subsequent norovirus season, though a few individuals may acquire longer-lasting immunity. With these factors combined, one might think that immune selection pressure would be rather transient—only heavy at the end of a season—and that an evolutionarily stable strategy for norovirus might be to wait out the summer low season and attack again when population immunity has waned. This is not what Baric and colleagues have found.

## Findings of the New Study

Instead, their findings suggest (1) that noroviruses are under heavy selective pressure and (2) that the norovirus capsid, which contains both antigenic sites and carbohydrate binding ligands, seems to have been finely tuned to evolve. The P2 region of the capsid is attached to the virus shell by a flexible hinge, so—with minor genetic tweaking—the virus can nuzzle up to a range of HBGA sites. This domain is evolving at a faster rate than regions not coding for surface residues.

But perhaps Baric's most compelling evidence that the virus evolves in the face of human immunity comes in the form of serology (since noroviruses can't be grown in cell culture, blocking assays are used as an alternative to neutralising antibody experiments). Pre-epidemic anti-serum binds poorly to post-epidemic virus-like particles; this observation was most extreme for the 2002–2003 epidemic, which by many accounts [6,7] was the most severe epidemic recorded. That year's strain appears to have had a novel surface antigen, effectively leaving the whole population susceptible. Once again, as with influenza, it appears that noroviruses may undergo genetic drift, punctuated by a shift every three years or so. The trade-off between replication fitness and evasion of the immune response may underlie the so-called “epochal shifts”. Interestingly, these shifts seem to be occurring more frequently now, with epidemic variants identified in 2002, 2004, and 2005. This 2007–2008 winter may have been epidemic in the United Kingdom, where extensive hospital ward outbreaks occurred [8], and perhaps in other countries as well. Could it be that norovirus has pushed itself into an evolutionary corner: having caused widespread infection, and therefore higher levels of population immunity, might it need to evolve faster to persist?

There are, however, important differences between influenza and norovirus evolution and epidemiology. Although GII.4 viruses predominate, they do so within a large population of co-circulating genotypes. In contrast, a new influenza subtype generally replaces existing types. Following antigenic shift, replaced influenza types tend to go extinct, while usurped norovirus variants continue to circulate at low levels. Such differences are probably driven by the short-lived non-sterilising immunity to norovirus compared with the longer-term protection to influenza antigens. Indeed, the other norovirus genotypes may well be operating under different selective pressure than GII.4.

## What Next?

Baric and colleagues' landmark paper sets forth a wide research agenda for norovirus vaccinology, virology, and epidemiology. The authors are bullish that their findings take us towards the development of norovirus vaccines. This is undoubtedly true—though it is unlikely that a major pharmaceutical company will invest the massive sums required to bring to market a product for such an antigenically diverse virus that confers short-lived immunity and is perceived to cause a relatively low burden of disease. We think this lack of investment in developing a vaccine would be unfortunate; vaccination targeted at vulnerable populations and health care workers could mitigate the most severe health and economic consequences caused by noroviruses. Formulation of a norovirus vaccine would be challenging, but tools developed for understanding influenza evolution could prove useful. For example, data generated from Baric and colleagues' serological experiments lend themselves to fascinating antigenic mapping methods used to quantify and visualise antigenic differences between circulating influenza strains [9], and may prove effective in predicting norovirus evolution.

What is really needed—but has been lacking—is a good in vitro cell culture system. Such a system would facilitate investigation of the relationship between antigen and receptor and how antibody interferes with binding. The finding that secretor-negative individuals are still susceptible to infection suggests that the HBGAs are only one link in a binding hierarchy.

An area absent from Baric and colleagues' paper is a discussion of the seasonality of norovirus. Like influenza, noroviruses exhibit a strong wintertime seasonality (see Figure 1 and [10] for a discussion of flu seasonality), and new variants disseminate rapidly. But unlike flu, noroviruses do not disappear during summer, nor do they have animal hosts acting as a reservoir for frequent re-introductions. The environmental and host behavioural factors that may influence norovirus seasonality are not understood. But such factors are likely to interact with herd immunity in a complex way. For example, environmental factors (such as lower temperature and diminished ultraviolet light) may increase the virus' transmission potential, exacerbated by high winter-time hospital occupancy; this in turn may trigger a seasonal epidemic, which results in high levels of population immunity. At the springtime end of the season, population immunity, and hence selective pressure, would be at its highest. Indeed, it is in the warmer months that new epidemic variants often emerge. Baric and colleagues' article arrives at the height of the norovirus season. But as spring comes and the annual epidemic wanes, we should remain vigilant. It is then that the next pandemic strain is likely to emerge.

**Figure 1:**
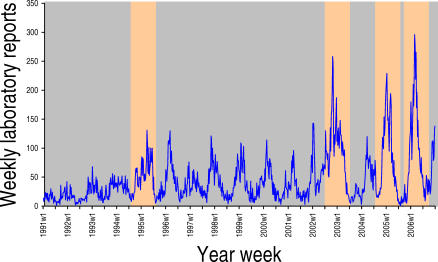
Laboratory Reports of Norovirus-Positive Specimens in England and Wales, 1991 to 2006 Season-associated novel antigen variants of GII.4 are highlighted in orange.

## Abbreviations

HBGA: histoblood group antigen
